# LncRNA HOXC-AS3 accelerates malignant proliferation of cervical cancer cells via stabilizing KDM5B

**DOI:** 10.1007/s00432-024-05799-y

**Published:** 2024-06-06

**Authors:** Jie Li, Fang Hou, Zhenghua Teng, Weiwei Xia, Jie Peng

**Affiliations:** Department of Obstetrics and Gynecology, Suzhou Wuzhong People’s Hospital, Jiangsu Province, No. 61, Dongwu North Road, Suzhou City, 215128 China

**Keywords:** Cervical cancer, LncRNA HOXC-AS3, KDM5B, Proliferation, TRIM37

## Abstract

**Background:**

Cervical cancer (CC) is a common malignancy amongst women globally. Ubiquitination plays a dual role in the occurrence and development of cancers. This study analyzed the mechanism of long noncoding RNA HOXC cluster antisense RNA 3 (lncRNA HOXC-AS3) in malignant proliferation of CC cells via mediating ubiquitination of lysine demethylase 5B (KDM5B/JARID1B).

**Methods:**

The expression patterns of lncRNA HOXC-AS3 and KDM5B were measured by real-time quantitative polymerase chain reaction or Western blot analysis. After transfection with lncRNA HOXC-AS3 siRNA and pcDNA3.1-KDM5B, proliferation of CC cells was assessed by the cell counting kit-8, colony formation, and 5-Ethynyl-2’-deoxyuridine staining assays. The xenograft tumor model was established to confirm the impact of lncRNA HOXC-AS3 on CC cell proliferation in vivo by measuring tumor size and weight and the immunohistochemistry assay. The subcellular location of lncRNA HOXC-AS3 and the binding of lncRNA HOXC-AS3 to KDM5B were analyzed. After treatment of lncRNA HOXC-AS3 siRNA or MG132, the protein and ubiquitination levels of KDM5B were determined. Thereafter, the interaction and the subcellular co-location of tripartite motif-containing 37 (TRIM37) and KDM5B were analyzed by the co-immunoprecipitation and immunofluorescence assays.

**Results:**

LncRNA HOXC-AS3 and KDM5B were upregulated in CC tissues and cells. Depletion of lncRNA HOXC-AS3 repressed CC cell proliferation and in vivo tumor growth. Mechanically, lncRNA HOXC-AS3 located in the nucleus directly bound to KDM5B, inhibited TRIM37-mediated ubiquitination of KDM5B, and upregulated the protein levels of KDM5B. KDM5B overexpression attenuated the inhibitory role of silencing lncRNA HOXC-AS3 in CC cell proliferation in vivo and in vitro.

**Conclusion:**

Nucleus-located lncRNA HOXC-AS3 facilitated malignant proliferation of CC cells via stabilization of KDM5B protein levels.

## Background

Cervical cancer (CC), primarily including cervical squamous cell carcinoma (over 70% of all CC cases) and cervical adenocarcinoma (over 25% of all CC cases), is recognized as a commonly diagnosed malignant tumor in gynecology associated with a high occurrence for the reason of persistent infections with high risk human papillomavirus (HPV), threatening the health status of women all over the world (Johnson et al. 2019). In 2020, an estimated number of over 604,127 new cases of CC are diagnosed and over 341,831 deaths occur as a result of this debilitating malignancy (Sung et al. 2021). Despite the ongoing implements of treatment modalities, such as surgery, radiotherapy, and chemotherapy, the resistance, metastasis, and recurrence of CC lead to unsatisfactory overall survival rates of CC patients (Feng et al. 2020; Adiga et al. 2021). Therefore, an in-depth investigation of malignant proliferation of CC cells is instrumental in understanding the occurrence and progression of CC.

Long noncoding RNAs (lncRNAs), a cluster of regulatory noncoding RNAs consisting of transcripts over 200 nucleotides, mediate gene expression in a transcriptional and/or post-transcriptional manner and exhibit biological functions in a myriad of cellular processes, such as cell cycle, differentiation, and metabolism (Bridges et al. 2021). In addition, lncRNAs are evidenced to participate in the regulation of cancer-related cell signaling pathways, proliferation, migration, metastasis, and drug resistance, thus affecting cancer progression (Li et al. 2016; Peng et al. 2017). As an example of these lncRNAs, lncRNA HOXC cluster antisense RNA 3 (HOXC-AS3) has been reported to facilitate progression of gastric and ovarian cancer types by modulating cell proliferation (Zhang et al. 2018; Yang et al. 2021). More importantly, higher level of lncRNA HOXC-AS3 accelerates the progression of CC and portends poorer prognosis (Zhao et al. 2021a). Nonetheless, the potential molecular activity of lncRNA HOXC-AS3 in proliferation of CC cells remains largely unknown.

Lysine demethylase 5B (KDM5B/JARID1B) is a well-identified histone demethylase that catalyzes histone 3 lysine 4 (H3K4) demethylation, possessing a wide range of epigenetic regulations on cell phenotypes during normal cell growth or carcinogenesis of cells (Xhabija and Kidder 2019). KDM5B is robustly expressed and endowed with oncogenic activity in multiple cancers, such as prostate, hepatocellular, and breast cancers (Gong et al. 2018; Zhang et al. 2019; Yang et al. 2020). Besides, KDM5B knockdown can restrain CC cell growth (Zhou et al. 2017). Here, bioinformatic results revealed the binding association between lncRNA HOXC-AS3 and KDM5B. Further, lncRNA HOXC-AS3 can regulate E3 ubiquitin ligase-mediated ubiquitination via binding to its target genes (Su et al. 2022). Ubiquitination is defined as a post-translational modification to regulate the conjugated protein via the attachment of ubiquitin molecules to the protein and reduce protein stability by trafficking ubiquitin-marked protein to the proteasome or lysosome (Gu and Jan Fada 2020). Ubiquitination exerts both promotive and suppressive roles in cancers by regulating oncogenic and tumor-suppressive proteins (Mansour 2018). As a E3 ubiquitin ligase, tripartite motif-containing 37 (TRIM37) is confirmed to have an impact on the progression of CC (Song et al. 2021). Besides, the Ubibrowser database predicted that TRIM37 is one of the ubiquitin enzymes that can interact with KDM5B. Consequently, we conceived a hypothesis that lncRNA HOXC-AS3 may affect proliferation of CC cells with the assistance of KDM5B and TRIM37 and we hope this study can provide a new therapeutic strategy for treating CC.

## Materials and methods

### Sample collection

The study was implemented with the approval of the Ethics Committee of Suzhou Wuzhong People’s Hospital (Approval number: 2021WZ-471), and all recruited patients signed an informed consent form. A total of 45 CC patients who were diagnosed in Suzhou Wuzhong People’s Hospital from 2018 to 2021 were recruited in this study (age range: 37–62 years old; the average age: 50.36 years old). Cancer tissues and para-cancerous tissues of CC patients were collected and then immediately kept in liquid nitrogen. The inclusion criteria were as follows: (1) Pathologically diagnosed with CC; (2) Without previous treatment history of radiotherapy, chemotherapy, or other adjuvant managements. The exclusion criteria were as follows: (1) With distant metastasis; (2) With other types of cancers; (3) During pregnancy or breastfeeding.

### Cell culture

Normal cervical epithelial cell line Ect1/E6E7 cells (ATCC, Manassas, VA, USA) were cultured in a Roswell Park Memorial Institute (RPMI)-1640 liquid culture medium (ThermoFisher Scientific, Shanghai, China) and CC cell lines C-33A (bio-69458), HeLa (bio-68123), SiHa (bio-69163), and CaSki cells (bio-69460) (ATCC, Manassas, VA, USA) were cultured in a Dulbecco’s modified Eagle medium (DMEM)-F12 liquid culture medium (ThermoFisher Scientific, Shanghai, China), with a temperature of 37℃ and an air condition of 5% CO_2_. Both cell media were added with 10% fetal bovine serum (ThermoFisher Scientific, Shanghai, China) and 100 U/mL penicillin and streptomycin (Beyotime, Shanghai, China) in advance.

### Cell treatment

LncRNA HOXC-AS3 siRNAs (si‑HOXC-AS3‑1, si‑HOXC-AS3‑2, and si‑HOXC-AS3‑3), TRIM37 siRNAs (si‑TRIM37‑1, si‑TRIM37‑2, and si‑TRIM37‑3), negative control (si‑NC), were designed and synthesized by Shanghai GenePharma Co., Ltd and their sequences are shown in Table 1. pcDNA3.1-HOXC-AS3 (HOXC-AS3), pcDNA3.1-KDM5B (KDM5B), and pcDNA3.1-empty vector (NC) were also obtained from GenePharma (Shanghai, China). When HeLa or CaSki cells grew to approximately 70% confluence, the expression vectors or sequences were transfected into these cells using Lipofectamine 3000 (Invitrogen, Carlsbad, CA, USA). Then, real-time quantitative polymerase chain reaction (RT-qPCR) and Western blot (WB) assays were employed to measure the transfection efficiency.

**Table 1:** siRNA sequences

siRNAs	SS sequence	AS sequence
si‑HOXC-AS3‑1	GGGAGCAUGUUUGAAAGAAGA	UUCUUUCAAACAUGCUCCCAG
si‑HOXC-AS3‑2	GGAGGAGUCACGUAUCACACG	UGUGAUACGUGACUCCUCCAA
si‑HOXC-AS3‑3	AGAAGACAGACCAGUCUAUUU	AUAGACUGGUCUGUCUUCUUU
si‑TRIM37‑1	CUGUGUUGUUUCAGCUGUAUU	UACAGCUGAAACAACACAGUU
si‑TRIM37‑2	GCUGCACAGACUAGUUAUAUC	UAUAACUAGUCUGUGCAGCUU
si‑TRIM37‑3	GACUAGUUAUAUCCAACAAAU	UUGUUGGAUAUAACUAGUCUG
si-NC	GGAGGGCAGUUCAGUACAUUG	CUGCAGAACUCUACGUCAUCG

### Cell counting kit-8 (CCK-8) method

Cell suspensions in different groups were diluted and seeded in 96-well plates at a density of 1 × 10^3^ cells/well, and were divided into 4 groups (0 h, 24 h, 48 h, 72 h) based on the incubation time with 3 repetitive wells for each group. The plates were incubated at 37℃ with 5% CO_2_, and 10 μL of CCK-8 solution (Sigma, St. Louis, MO, USA) was added to each well at each specified time point, after which the plates were incubated for 4 h. Then, the absorbance under 450 nm wavelength was determined by a microplate reader (Bio-Rad, Hercules, CA, USA).

### Colony formation assay

Cells in different groups were seeded in 6-well plates at a density of 1 × 10^3^ cells/well for 2 weeks of culture, fixed with formaldehyde, and stained with crystal violet for 10 min. After 3 times of washing with phosphate buffered-saline (PBS), the stained colonies (composed of over 50 cells) were imaged and counted using an optical microscope (Olympus, Tokyo, Japan).

### 5-ethynyl-2′ deoxyuridine (EdU) staining

Cells in different groups were placed at a density of 1 × 10^5^ cells/well in 96-well plates supplemented with 50 μM EdU medium (100 μL/well; ThermoFisher Scientific, Shanghai, China) for 2-h incubation (37 °C and 5% CO_2_), and cell proliferation was determined in strict accordance with the provided instructions of the EdU binding assay kit (Guangzhou RiboBio Co., Ltd., Guangzhou, Guangdong, China). Thereafter, cells were treated with 4% paraformaldehyde for 30 min and stained with 100 μL Apollo staining solution (Guangzhou RiboBio Co., Ltd.) for 30 min, and the cell nuclei were stained with 4′,6-diamidino-2-phenylindole (DAPI) for 30 min. The stained cells were observed using a fluorescence microscope (Olympus, Tokyo, Japan), and the final results were expressed as percentages.

### Bioinformatic analysis

LncRNA HOXC-AS3 expression levels in cervical squamous cell carcinoma were predicted via the UALCAN database (https://ualcan.path.uab.edu/analysis.html) (Chandrashekar et al. 2022). The possible interaction between lncRNA HOXC-AS3 and KDM5B was analyzed via the RNAInter database (http://www.rnainter.org/) (Kang et al. 2022), and the E3 ubiquitin ligases that may regulate KDM5B were predicted via the Ubibrowser database (http://ubibrowser.bio-it.cn/ubibrowser_v3) (Li et al. 2017).

### Nuclear/cytoplasmic fractionation assay

Firstly, the subcellular fractionation was conducted using a PARIS™ kit (Ambion, Austin, Texas, USA). The collected cells were rinsed with PBS, placed on ice, resuspended in 500 μL pre-cooled cell isolation buffer, and dissolved on ice for 10 min. After centrifugation at 300 × *g* for 5 min, the supernatants (cytoplasm) were isolated from the precipitates (nucleus), and the isolated supernatants were mixed thoroughly with an equal amount of 2 × Lysis/Binding solution, to prevent the degradation of RNA, followed by the addition of an equal amount of absolute alcohol. Thereafter, the cocktail was added to a filter candle, and after several washes of the filter candle, the liquid was discarded, and the RNA (the dissolved cytoplasmic RNA) on the filter was obtained and centrifugated for 30 s. The nuclear precipitates (the nuclear RNA) were collected following the above-described processes. LncRNA HOXC-AS3 expression levels in cytoplasm and nuclei were determined using RT-qPCR.

### RNA fluorescence in situ hybridization (FISH) method

The location of lncRNA HOXC-AS3 in cells was determined using the FISH method. Cells were detached by 0.25% trypsin, and the medium was abandoned after 24-h culture. Cells were washed with PBS twice, fixed with 4% paraformaldehyde, washed with PBS three times, and stained with 5(6)-carboxyfluorescein-labeled probes that targeted lncRNA HOXC-AS3 (GenePharma) at 4 °C for 15 h, and the cell nuclei were stained with DAPI (Beyotime). The fluorescent signals were observed using a Leica SP5 confocal microscope (Leica Microsystems, Mannheim, Baden-Württemberg, Germany).

### RNA immunoprecipitation (RIP) assay

Cells were lysed in the lysis buffer (50 mM Tris–HCl, 2.5 mM EDTA, 130 mM NaCl, and 1% NP-40) containing ribonuclease and protease inhibitor (ThermoFisher Scientific, Shanghai, China). A portion of the supernatants were used as the input and the other portion of the supernatants were incubated with primary antibody against KDM5B (ab181089, Abcam, Cambridge, MA, USA) or antibody against IgG (ab172730, Abcam, Cambridge, MA, USA) at 4 ℃ overnight. Samples conjugated with antibodies were incubated with protein-A Sepharose (Sigma, St. Louis, MO, USA) at 4 ℃ for 2 h, washed with washing buffer, and incubated with protease K (Sangon Biotech Co., Ltd., Shanghai, China) for 30 min. The total RNA was extracted using the TRIzol solution (Invitrogen, Carlsbad, CA, USA) for RT-qPCR.

### RNA pull-down assay

Biotin-labeled HOXC-AS3 and antisense lncRNA HOXC-AS3 transcripts were obtained from GenePharma. After cell transfection, the cell lysates were yielded, and a portion of the lysates were used as the input and the other portion of the lysates were incubated with biotinylated lncRNA HOXC-AS3 at 4℃ for 2 h. The RNA–protein compounds were further isolated using Dynabeads M-280 streptavidin (Invitrogen, Carlsbad, CA, USA). The beads were incubated with biotinylated lncRNA HOXC-AS3 and antisense HOXC-AS3 for 3 h and washed with washing buffer. KDM5B antibody (ab181089, Abcam, Cambridge, MA, USA) was used for WB assay to examine the recruited protein.

### Co-immunoprecipitation (Co-IP) assay

Cells were lysed in the lysis buffer (1 mM EDTA, 50 mM Tris–HCl, 200 mM NaCl, and 1% Triton X-100) containing protease inhibitor. Protein samples were examined by the bicinchoninic acid (BCA) assay kit to determine protein concentrations. Protein was incubated with the antibody against TRIM37 (ab264190, Abcam, Cambridge, MA, USA) or IgG (ab172730, Abcam, Cambridge, MA, USA) at 4 ℃ overnight, added with protein-A Dynabeads (ThermoFisher Scientific, Shanghai, China) at 4 ℃ for 3 h, washed with lysis buffer, eluted using sodium dodecyl sulfate (SDS) loading buffer, and used for WB assay.

### Immunofluorescence assay

Cells were rinsed with pre-cooled PBS twice, fixed with 4% paraformaldehyde for 30 min at room temperature, washed again with PBS twice, followed by an addition of 0.1% Triton X-100 at room temperature. After approximate 10 min, cells were added with normal goat serum, blocked at room temperature for 1 to 2 h, incubated with antibodies against KDM5B (ab244220, Abcam, Cambridge, MA, USA) and against TRIM37 (ab238156, Abcam) at 4 ℃ overnight, washed with PBS-Tween-20 three times (10 min for each time), stained with DAPI for 3 to 5 min, washed with PBS 3 to 5 times, and mounted in a mounting medium. Cell confocal images were captured using a Leica SP5 confocal microscope (Leica Microsystems).

### MG132 treatment

Cells were treated with 5 μM MG132 (a protease inhibitor; MCE, Monmouth Junction, NJ, USA) for 4 h, collected, and lysed using lysis buffer. Cell lysates were incubated with KDM5B antibody (ab181089, Abcam, Cambridge, MA, USA) or IgG antibody (ab133470, Abcam, Cambridge, MA, USA), added with protein A/G plus-Agarose beads, and incubated at 4℃ overnight. Afterwards, the ubiquitination levels of KDM5B were determined by WB assay.

### Xenograft experiment

The animal experimental program was ratified by the Ethic Committee of Suzhou Wuzhong People’s Hospital (Approval number: 2021WZ-163), and the use, care, and handling procedures on animals complied with the Guide for the Care and Use of Laboratory Animals published by the National Institutes of Health (National Research Council (US) 2011). BALB/c nude mice (age: 4 to 6 weeks old; weight: 16 to 18 g; Vital River Laboratory Animal Technology Co., Ltd., Beijing, China; license number: SYXK (Beijing) 2017–0033) were divided into 2 groups with 12 mice per group, housed in an environment of 24 °C, 50% humidity, and 12-h day/night cycles, and had free access to water and food. To establish the xenograft model, HOXC-AS3-knockdown lentivirus (sh-HOXC-AS3) was used to infect CaSki cells. The infected CaSki cells were purified by 5 μg/mL puromycin to obtain cells with stable HOXC-AS3 knockdown. Each mouse was subcutaneously injected with the stably infected CaSki cells (N = 3 × 10^6^ cells; in 100 μL aseptic PBS solution) on the right side of the mouse. Then, tumor growth was detected for 4 consecutive weeks. Tumor volume was calculated as length (mm) × width (mm^2^)/2. After 4 weeks, nude mice were euthanatized by an intraperitoneal injection of ≥ 100 mg/kg pentobarbital sodium, and their tumors were resected and weighted. Then, 6 mice from each group were randomly selected for immunohistochemistry (IHC) and the remaining 6 mice were used for molecular experiments.

### Immunohistochemistry (IHC)

The resected tissues were embedded in paraffin, sliced (4-μm thick), dewaxed using xylene, hydrated using gradient ethanol, and blocked with 3% H_2_O_2_ for 10 min to inactivate endogenous peroxidase. Microwave restore (pH = 6.0, 15 min) was performed using 0.01 mol/L sodium citrate buffer. After blockade with 5% bovine serum albumin (Beyotime) for 20 min, slices were incubated with antibody against Ki67 (ab15580, Abcam, Cambridge, MA, USA) at 4℃ overnight and with second antibody against IgG (ab6721, Abcam, Cambridge, MA, USA) for 30 min at room temperature, washed with PBS, and developed using 3,3′-diaminobenzidine. After counterstaining with hematoxylin, slices were dehydrated to transparency and observed using a microscope (Olympus, Tokyo, Japan).

### RT-qPCR

The total RNA in tissue samples and cells was separated using the TRIzol solution (Invitrogen, Carlsbad, CA, USA). A260 and A280 were detected using a Nano-Drop ND-1000 spectrophotometer to analyze the concentration and purity of RNA. The extracted RNA was processed by a PrimeScript RT kit (Takara, Tokyo, Japan) to acquire the cDNA. qPCR reaction was performed on the ABI7500 real-time PCR system using SYBR green (Takara, Tokyo, Japan). With GAPDH as the internal standard, the relative gene expressions were analyzed using the 2^−ΔΔCt^ method (Livak and Schmittgen 2001), and PCR primer of each gene is presented in Table 2. The experiment was repeated three times.

**Table 2:** PCR primer sequences

Name	Sequence (5′-3′)
HOXC-AS3	F: GTGGAGTAACAGCGCCATCT
	R: CGGGTTTTGTTGCGTCTTGT
KDM5B	F: ACCCCTTCGCTTTCATCCAC
	R: CAGTCTCTGGATACGTGGCG
TRIM37	F: ATGAACAGAGCGTGGAGAGC
	R: GTGCATCCCGCAATTTCTCC
GAPDH	F: GATGCTGGCGCTGAGTACG
	R: GCTAAGCAGTTGGTGGTGC

### WB assay

The radioimmunoprecipitation assay lysis buffer (Sigma, St. Louis, MO, USA) was applied to isolate the total protein from tissue samples and cells, and the BCA assay kit was used to determine the protein concentrations. Protein samples were processed by 10% SDS–polyacrylamide gelelectrophoresis and transferred onto nitrocellulose membranes. After the blockade with 5% defatted milk at room temperature for 2 h, membranes were incubated with the following primary antibodies: KDM5B (ab181089, 1:1000, Abcam, Cambridge, MA, USA), TRIM37 (ab264190, 1:2000, Abcam, Cambridge, MA, USA), and GAPDH (ab9485, 1:2500, Abcam, Cambridge, MA, USA) at 4 ℃ overnight, and with goat anti-rabbit immunoglobulin G (horseradish peroxidase) antibody (ab205718, 1:2000, Abcam, Cambridge, MA, USA) at room temperature for 1 h after being washed with PBS thrice. Lastly, protein levels were measured using the enhanced-chemiluminescence reagent (Millipore, Billerica, MA, USA) and analyzed using ImageJ software (version 1.48, National Institutes of Health, Bethesda, MD, USA).

### Statistical approaches

Analysis and graphing on statistical data were conducted via SPSS 21.0 (IBM SPSS Statistics, Chicago, IL, USA) and GraphPad Prism 8.0 (GraphPad Software Inc., San Diego, CA, USA). Measurement data were shown as normal distribution with uniform variance, and statistical differences between 2 groups were examined using the *t*-test while statistical differences among multiple groups were examined using one-way analysis of variance (ANOVA) or two-way ANOVA, after that, Tukey’s multiple comparison test was used for the post-test. *p* < 0.05 indicated statistical difference and *p* < 0.01 indicated significantly statistical difference.

## Results

### LncRNA HOXC-AS3 expression levels are upregulated in CC

**Fig. 1:**
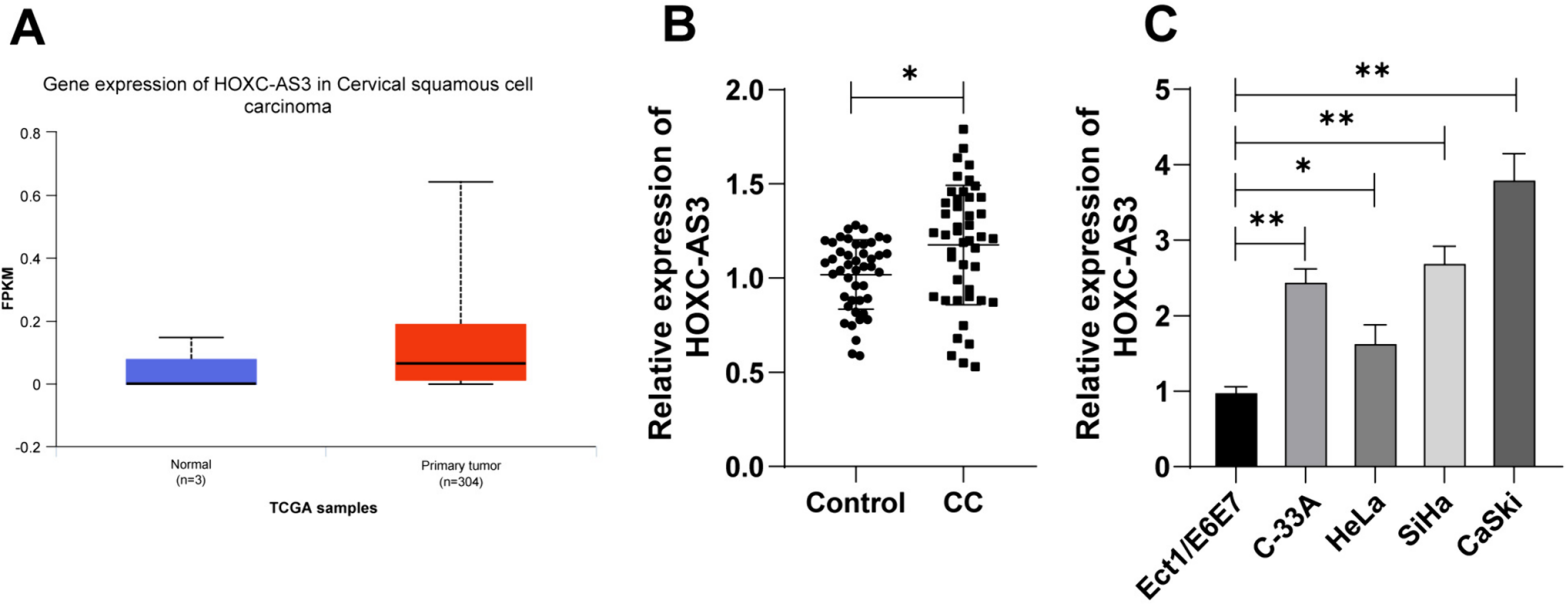
HOXC-AS3 expression levels were upregulated in CC. **A** HOXC-AS3 expression levels in cervical squamous cell carcinoma were analyzed via the UALCAN database. **B** HOXC-AS3 expression levels in 45 pairs of cancer tissues and para-cancerous tissues of CC patients were detected via RT-qPCR, *N* = 45; **C** HOXC-AS3 expression levels in normal cervical epithelial cell line (Ect1/E6E7 cells) and in CC cell lines (C33A, Hela, SiHa, and CaSki cells) were examined via RT-qPCR. Cell experiments were independently repeated 3 times. Data in panel **C** were shown as mean ± standard deviation; data comparisons in panel **B** were tested via the *t *test; data comparisons in panel C were tested via one-way ANOVA, followed Tukey’s multiple comparison test. *p < 0.05; **p < 0.01

A previous study has reported the upregulation of lncRNA HOXC-AS3 in CC (Zhao et al. 2021a). To start with, lncRNA HOXC-AS3 expression levels in normal tissues and CESC tissues were analyzed by the UALCAN database, and the results showed that lncRNA HOXC-AS3 was overexpressed in cervical squamous cell carcinoma tissues compared with normal tissues (Fig. 1A). Subsequently, lncRNA HOXC-AS3 expression levels in tissues collected from patients were detected, and compared with the para-cancerous tissues, lncRNA HOXC-AS3 expression levels were higher in CC tissues (*p* < 0.05, Fig. 1B). To explore the clinical significance of HOXC-AS3 in CC, we assigned CC patients into the HOXC-AS3 high-expression group and HOXC-AS3 low-expression group with the median expression level of HOXC-AS3 in CC tissues as the critical threshold (Xie et al. 2019). The correlation between HOXC-AS3 expression and clinicopathological characteristics and prognosis of CC patients was analyzed. The results showed that the expression of HOXC-AS3 was related to tumor size and international federation of gynecology and obstetrics (FIGO) stage (*p* < 0.05, Table 3). Further, lncRNA HOXC-AS3 expression levels in different cell lines were examined, and as a result, lncRNA HOXC-AS3 expression levels were increased in CC cell lines (C-33A, HeLa, SiHa, and CaSki cells) compared with Ect1/E6E7 cells (*p* < 0.05, Fig. 1C). Thus, the above data suggested the upregulation of lncRNA HOXC-AS3 in CC.

**Table 3:** Correlation between HOXC-AS3 and clinicopathological characteristics of cervical cancer patients

Characteristic	Number	HOXC-AS3		*P* value
	(*N* = 45)	Low expression (*N* = 23)	High expression (*N* = 22)	
Age				0.449
< 47	21	12	9	
≥ 47	24	11	13	
Pathological type				0.903
Squamous cell carcinoma	27	14	13	
Adenocarcinoma	18	9	9	
Tumor size				0.011
< 4 cm	25	17	8	
≥ 4 cm	20	6	14	
Lymph node metastasis				0.102
Negative	24	15	9	
Positive	21	8	13	
Differentiation				0.100
Well and moderately	22	14	8	
Poor	23	9	14	
FIGO stage				0.011
I	23	16	7	
II	22	7	15	

Data comparison is conducted using chi square test. FIGO, international federation of gynecology and obstetrics.* P* < 0.05 indicates statistical significance

### Silencing HOXC-AS3 retards malignant proliferation of CC cells

**Fig. 2:**
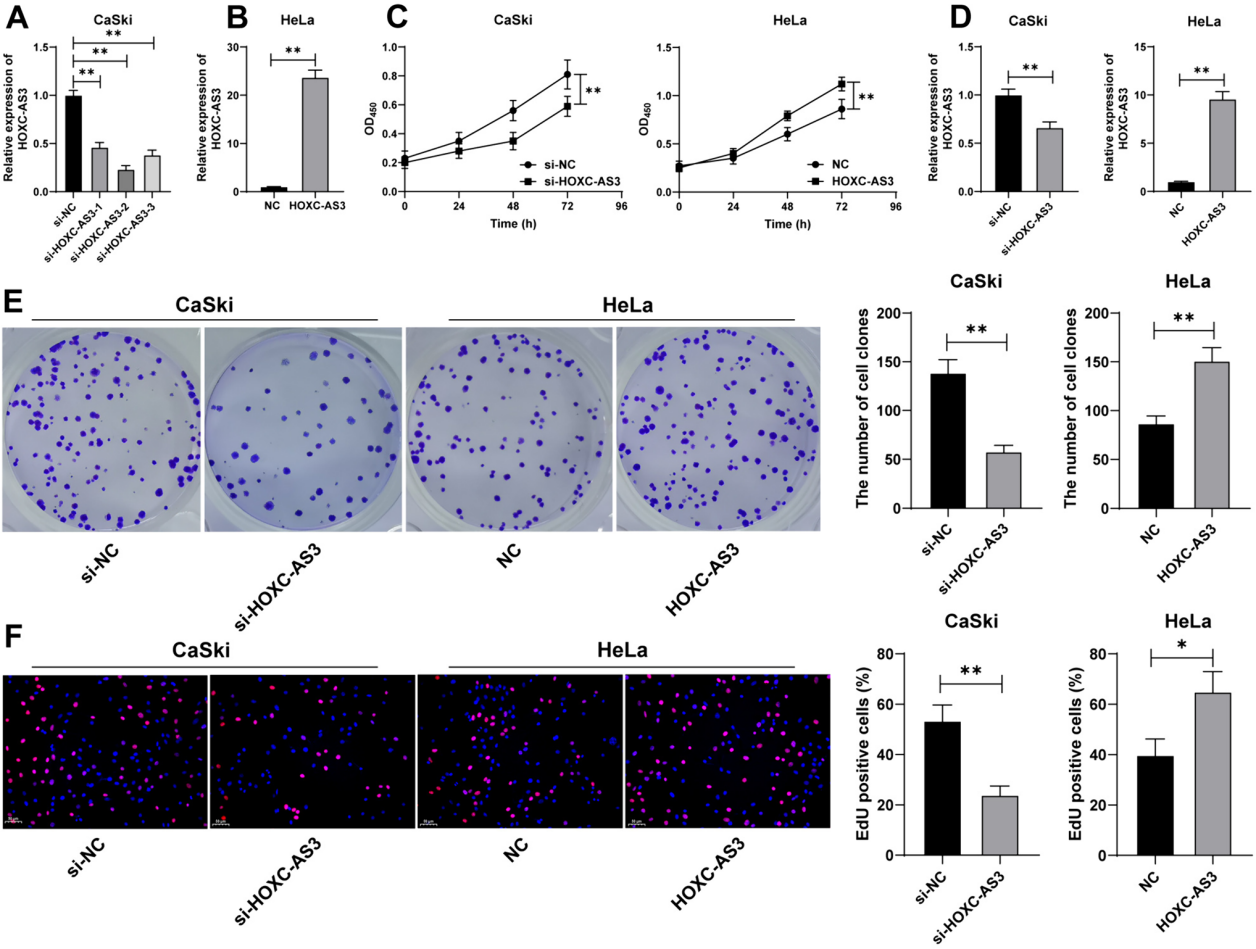
Silencing HOXC-AS3 retards malignant proliferation of CC cells. CaSki cells were transfected with HOXC-AS3 siRNA (si-HOXC-AS3), with NC siRNA (si-NC) as the negative control; HeLa cells were transfected with pcDNA3.1-HOXC-AS3 (HOXC-AS3), with pcDNA3.1-empty vector (NC) as the negative control. **A**, **B** HOXC-AS3 expression levels in CaSki and HeLa cells were detected via RT-qPCR. **C** Cell proliferation in different groups was determined via CCK-8 assay. **D** HOXC-AS3 expression in cells at 72 h was detected via RT-qPCR. **E**, **F** Cell proliferation in different groups was determined via colony formation and EdU assays. Cell experiments were independently repeated 3 times. Data were shown as mean ± standard deviation; data comparisons in panels **B**, **D**–**F** were tested via the *t *test; data comparisons in panel A were tested via one-way ANOVA and data in panel **C** were tested via two-way ANOVA, followed Tukey’s multiple comparison test. *p < 0.05; **p < 0.01

To determine the impact of HOXC-AS3 on proliferation of CC cells, lncRNA HOXC-AS3 siRNA (si-HOXC-AS3) was transfected into CaSki cells with relatively high expression of lncRNA HOXC-AS3 to downregulate lncRNA HOXC-AS3 expression levels in CaSki cells, and si-HOXC-AS3-2 with better silencing efficiency was chosen for the follow-up assays (*p* < 0.01, Fig. 2A). Meanwhile, HeLa cells with relatively low expression of lncRNA HOXC-AS3 were transfected with pcDNA3.1-HOXC-AS3 (HOXC-AS3) to increase cellular lncRNA HOXC-AS3 expression levels (*p* < 0.01, Fig. 2B). si-HOXC-AS3 reduced proliferation of CaSki cells while lncRNA HOXC-AS3 overexpression promoted proliferation of HeLa cells (*p* < 0.01, Fig. 2C, D). Besides, silencing lncRNA HOXC-AS3 reduced the number of colonies and the positive rate of EdU in cells while lncRNA HOXC-AS3 overexpression showed the opposite trends (*p* < 0.05, Fig. 2E, F). Collectively, these results indicated that silencing lncRNA HOXC-AS3 retards malignant proliferation of CC cells.

### LncRNA HOXC-AS3 diminishes KDM5B ubiquitination via binding to KDM5B

**Fig. 3:**
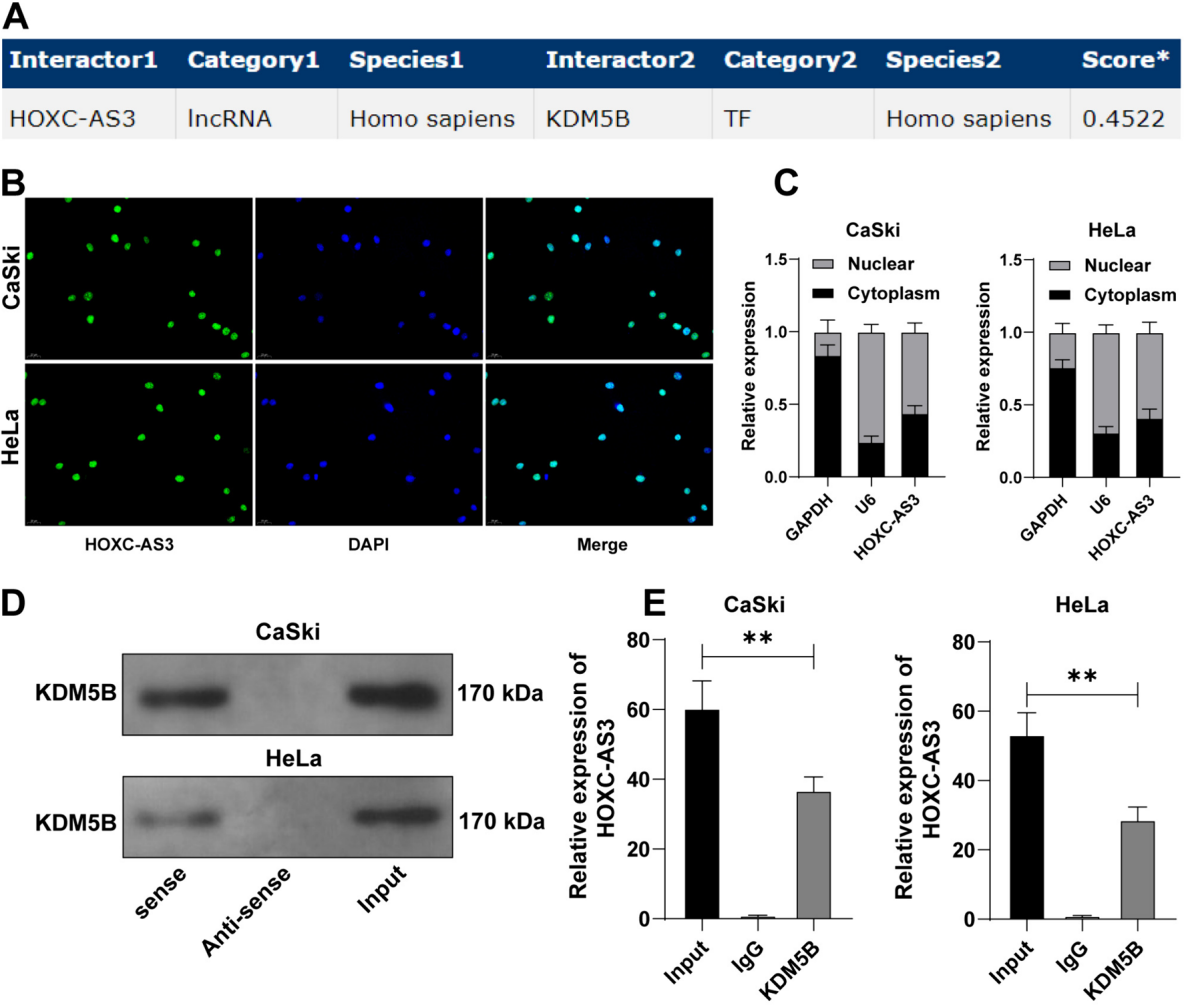
HOXC-AS3 directly binds to KDM5B. **A** The RNAInter database predicted the interaction between HOXC-AS3 and KDM5B. **B**, **C** The subcellular location of HOXC-AS3 was analyzed by the RNA FISH and nuclear/cytoplasmic fractionation assays. **D**, **E** The binding of HOXC-AS3 to KDM5B was analyzed by the RNA pull-down and RIP assays. Cell experiments were independently repeated 3 times. Data in panels were shown as mean ± standard deviation; data comparisons in panel **E** were tested via one-way ANOVA, followed Tukey’s multiple comparison test. **p < 0.01

Then, we attempted to probe the molecular mechanism of lncRNA HOXC-AS3 in CC. LncRNAs regulate the subcellular processes by directly binding to proteins (Marchese et al. 2017). Bioinformatic analysis (the RNAInter database) showed that lncRNA HOXC-AS3 may interact with KDM5B (Fig. 3A). Thus, we speculated that lncRNA HOXC-AS3 may participate in CC via regulating KDM5B. Next, we detected that lncRNA HOXC-AS3 was primarily expressed in the nucleus of CC cells (Fig. 3B, C). LncRNA HOXC-AS3 pull downed more KDM5B protein (Fig. 3D), and also, KDM5B antibody pulled down more lncRNA HOXC-AS3 (*p* < 0.05, Fig. 3E), suggesting lncRNA HOXC-AS3 directly bound to KDM5B in the nucleus.

**Fig. 4:**
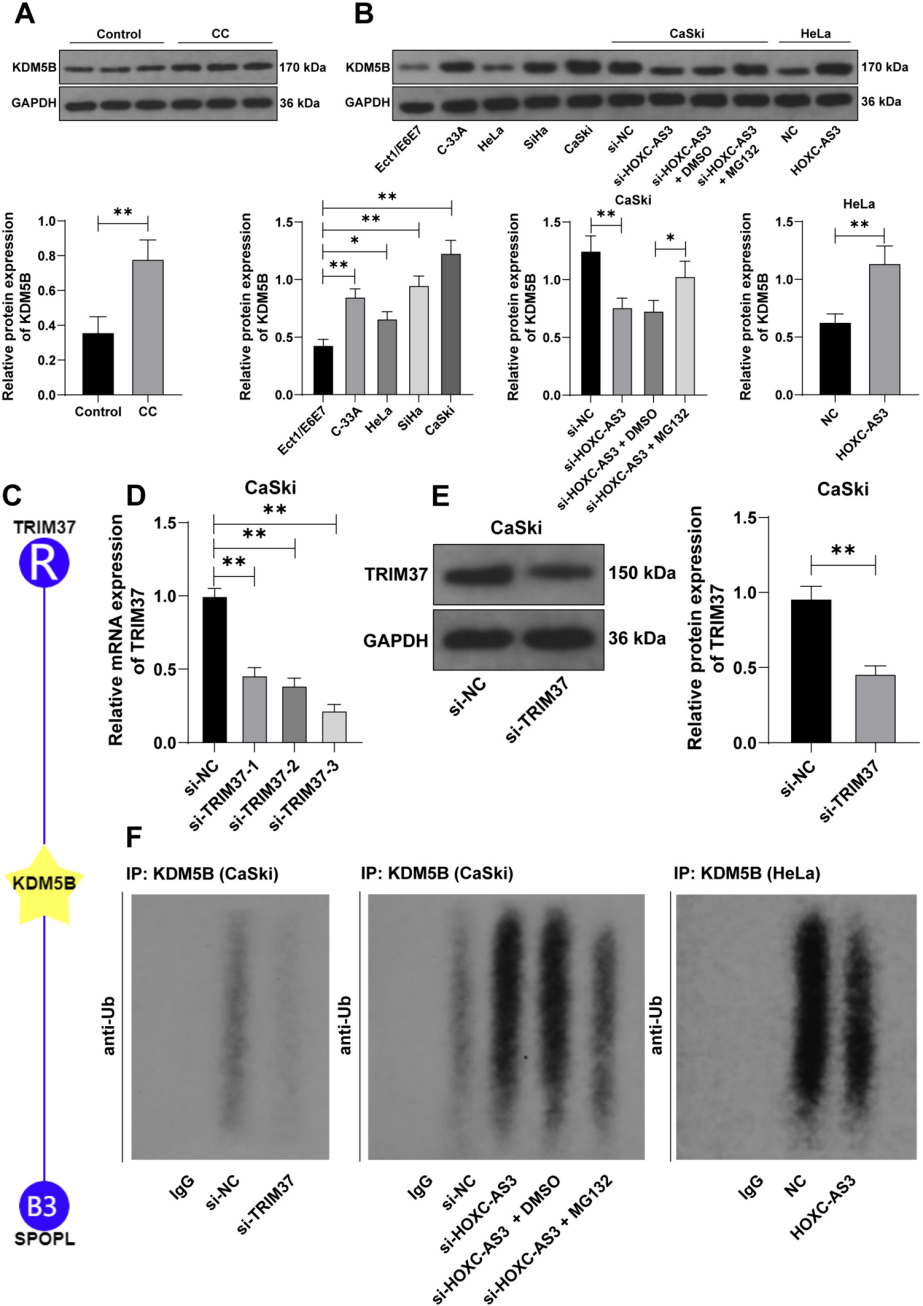
HOXC-AS3 diminishes KDM5B ubiquitination via binding to KDM5B. **A**, **B** KDM5B expression levels in CC tissues (*N* = 45; representative bands) and CC cells were detected via WB assay. **C** E3 ubiquitin ligases that can regulate KDM5B were predicted via the Ubibrowser database. CaSki cells were transfected with TRIM37 siRNA (si-TRIM37) with NC siRNA (si-NC) as the negative control. **D**, **E** TRIM37 expression levels in CaSki cells were detected via RT-qPCR and WB assays. **F** The ubiquitination levels of KDM5B cells in each group were detected via the ubiquitination assay. Cell experiments were independently repeated 3 times. Data were shown as mean ± standard deviation; data comparisons in panels **A**, **B** (right), and **E** were tested via the *t*-test; data comparisons in panels **B**, **D** were tested via one-way ANOVA, followed by Tukey’s multiple comparison test. *p < 0.05; **p < 0.01

Then, we investigated the effect of lncRNA HOXC-AS3 on regulating KDM5B expression levels. The results revealed that KDM5B expression levels were elevated in CC tissues and cells (*p* < 0.01, Fig. 4A), and lncRNA HOXC-AS3 knockdown decreased KDM5B protein levels whereas lncRNA HOXC-AS3 overexpression increased KDM5B protein levels (*p* < 0.05, Fig. 4B). Bioinformatic analysis (the Ubibrowser database) revealed that E3 ubiquitin ligases TRIM37 and SPOPL may promote KDM5B ubiquitination (Fig. 4C). Since previous study has reported the involvement of TRIM37 in CC (Song et al. 2021), TRIM37 was considered a focus of the subsequent experiments. Thereafter, our experimentation revealed that TRIM37 knockdown declined the ubiquitination levels of KDM5B (*p* < 0.01, Fig. 4D–F), and silencing lncRNA HOXC-AS3 increased KDM5B ubiquitination levels while lncRNA HOXC-AS3 overexpression diminished KDM5B ubiquitination levels (Fig. 4F). Besides, the proteasome inhibitor MG132 reversed the roles of silencing lncRNA HOXC-AS3 in the protein and ubiquitination levels of KDM5B (*p* < 0.05, Fig. 4B, F). Collectively, the above data suggested that lncRNA HOXC3-AS3 directly bound to KDM5B and stabilized KDM5B expression levels via inhibiting KDM5B ubiquitination.

### LncRNA HOXC-AS3 interferes with the interaction between TRIM37 and KDM5B

**Fig. 5:**
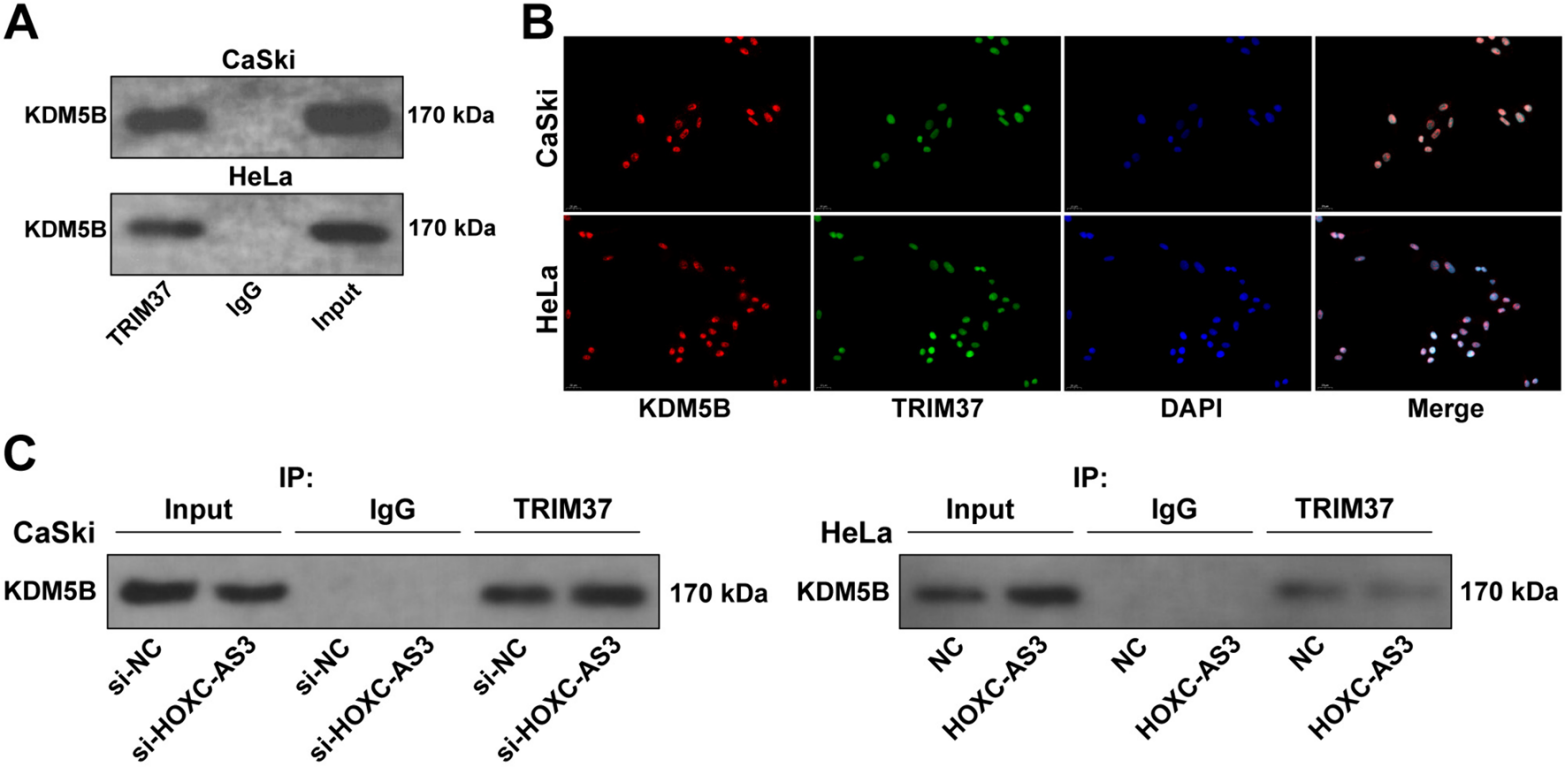
HOXC-AS3 interferes with the interaction between TRIM37 and KDM5B. **A** The interaction between KDM5B and TRIM37 was analyzed via the Co-IP assay. **B** The cellular co-location of KDM5B and TRIM37 was determined via the immunofluorescence assay. **C** The interaction between KDM5B and TRIM37 was analyzed via the Co-IP assay. Cell experiments were independently repeated 3 times

The molecular mechanism by which lncRNA HOXC-AS3 modulates KDM5B ubiquitination was further investigated. Based on the above results, we concluded that TRIM37 may affect KDM5B ubiquitination. Thereafter, our experiments revealed the direct interaction between TRIM37 and KDM5B (Fig. 5A) and the co-location of TRIM37 with KDM5B in cell nucleus (Fig. 5B). Furthermore, we aimed to figure out whether lncRNA HOXC-AS3 can affect the binding of TRIM37 to KDM5B. TRIM37 antibody pull downed more KDM5B in CaSki cells with low expression of lncRNA HOXC-AS3 while pull downed less KDM5B in CaSki cells with high expression of HOXC-AS3 (Fig. 5C), indicating that lncRNA HOXC-AS3 disrupted the interaction between TRIM37 and KDM5B.

### KDM5B overexpress neutralizes the function of lncRNA HOXC-AS3 knockdown in repressing proliferation of CC cells

**Fig. 6:**
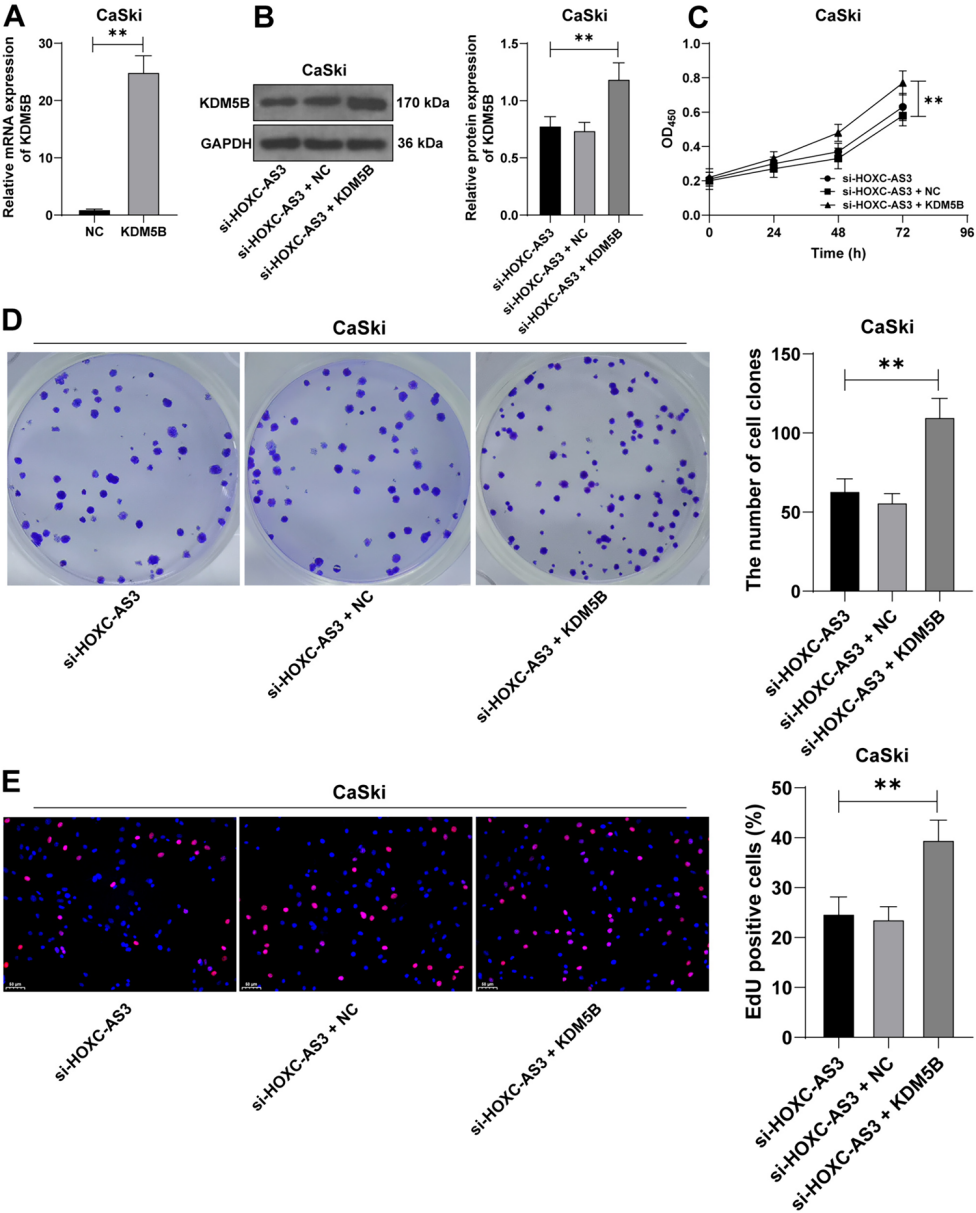
KDM5B overexpress neutralizes the function of HOXC-AS3 knockdown in repressing proliferation of CC cells. CaSki cells were transfected with pcDNA3.1-KDM5B (KDM5B) with pcDNA3.1-empty vector (NC) as the negative control. **A**, **B** KDM5B expression levels in CaSki cells were detected via RT-qPCR and WB assays. **C**–**E** Cell proliferation in different groups was determined via the CCK-8, colony formation, and EdU assays. Cell experiments were independently repeated 3 times. Data in panels were shown as mean ± standard deviation; data comparisons in panel **A** were tested via the *t *test; data comparisons in panels **B**, **D**, **E** were tested via one-way ANOVA and data in panel **C** were tested via two-way ANOVA, followed Tukey’s multiple comparison test. **p < 0.01

Subsequently, to explore the effect of KDM5B on proliferation of CC cells, KDM5B expression levels were upregulated in CaSki cells (*p* < 0.01, Fig. 6A, B), followed by combined treatment with with si-HOXC-AS3. The results showed that compared with the si-HOXC-AS3 + NC group, proliferative ability of cells was facilitated (*p* < 0.01, Fig. 6C), and the number of colonies and the positive rate of EdU of cells were elevated in the si-HOXC-AS3 + KDM5B group (*p* < 0.01, Fig. 6D, E). Hence, these results indicated that lncRNA HOXC-AS3 promoted proliferation of CC cells by modulating KDM5B.

### Silencing lncRNA HOXC-AS3 represses tumor growth by decreasing KDM5B expression levels

**Fig. 7:**
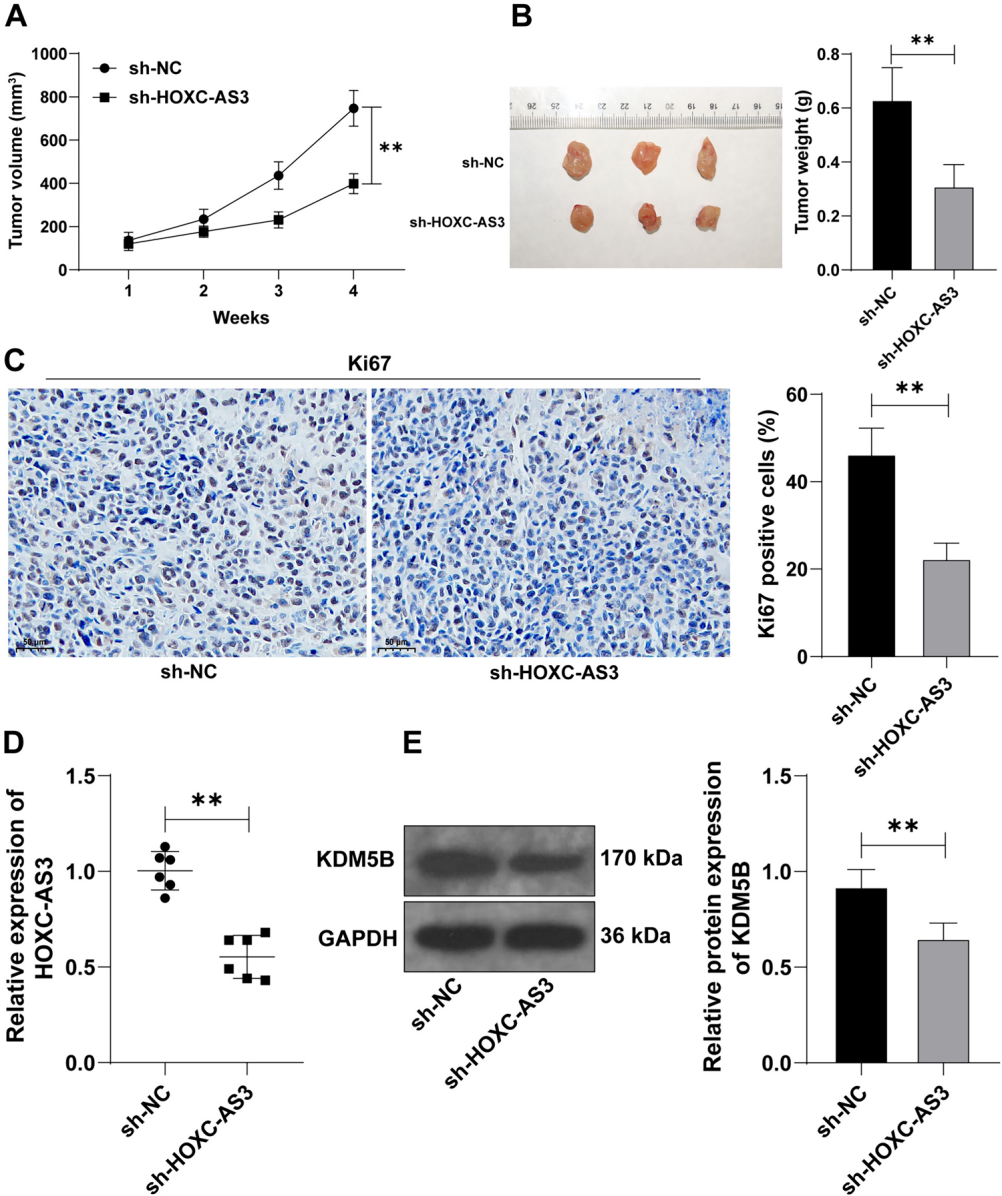
Silencing HOXC-AS3 represses tumor growth by decreasing KDM5B expression levels. CaSki cells with stable HOXC-AS3 knockdown (sh-HOXC-AS3) were used to establish the xenograft tumor model. **A** The tumor volume. **B** The representative picture and weight data of the resected tumors at the 4th week after animal euthanasia. **C** The positive rate of Ki67 in tumor tissues were measured via the IHC assay. **D** HOXC-AS3 expression levels in tumor tissues were detected via RT-qPCR. **E** KDM5B protein levels in tumor tissues were examined via the WB assay. *N* = 6; Cell experiments were independently repeated 3 times. Data in panels **A**–**C**, **E** were shown as mean ± standard deviation; data comparisons in panels **B**–**E** were tested via the *t *test; data comparisons in panel A were tested via two-way ANOVA, followed Tukey’s multiple comparison test. **p < 0.01

Lastly, we investigated the function of lncRNA HOXC-AS3/KDM5B in vivo. Compared with the sh-NC group, the tumor volume and weight were reduced in the sh-HOXC-AS3 group (*p* < 0.01, Fig. 7A, B), the positive rate of Ki67 was declined (*p* < 0.01, Fig. 7C), and the expression levels of lncRNA HOXC-AS3 and KDM5B were declined (*p* < 0.01, Fig. 7D, E). Altogether, the above results suggested that silencing lncRNA HOXC-AS3 retarded tumor growth via downregulating KDM5B.

## Discussion

To date, CC has been ranked as the fourth most common type of malignant tumors in females, accounting for high morbidity and CC-related mortality (Volkova et al. 2021). Accumulating studies have demonstrated that lncRNAs are implicated in the development of CC, including metastasis, metabolism, treatment resistance, and HPV regulation, and exhibit the potential as innovative biomarkers (Aalijahan and Ghorbian 2019; Galvao and Coimbra 2020; He et al. 2020). Additionally, the ubiquitination system plays a multifaceted role in cancers (Mansour 2018). In the present work, our observations initially disclosed that lncRNA HOXC-AS3 expedited malignant proliferation of CC cells by limiting TRIM37-mediated ubiquitination of KDM5B and upregulating KDM5B protein levels.

Mounting studies have highlighted the oncogenic activities of lncRNA HOXC-AS3 in multiple cancers. The increase of lncRNA HOXC-AS3 activated by glucose deprivation accelerates metabolic reprogramming of breast cancer cells in response to nutrient-stress conditions (Zhu et al. 2022). LncRNA HOXC-AS3 upregulation facilitates invasion and migration of non-small cell lung cancer cells (Wan et al. 2022; Zhang et al. 2022). In ovarian cancer, lncRNA HOXC-AS3 upregulation increases cell proliferation and inhibits apoptosis of ovarian cancer cells (Yang et al. 2021). In this work, our data showed that lncRNA HOXC-AS3 expression levels were increased in CC tissues and cells. Further, lncRNA HOXC-AS3 expression levels were downregulated in CaSki cells and upregulated in HeLa cells, respectively, and we disclosed that lncRNA HOXC-AS3 overexpression facilitated proliferation of CC cells while lncRNA HOXC-AS3 depletion exerted the reverse functions. In line with our results, Zhao et al. (2021a) elucidated that lncRNA HOXC-AS3 upregulation promotes tumorigenesis of CC via targeting the miR-105-5p/SOS1 axis, as manifested by strengthened proliferation and metastasis of CC cells. Nevertheless, Zhao et al. only reported the mechanism of HOXC-AS3 in the cytoplasm, which is a single mechanism. Our findings the first time reported the mechanism of HOXC-AS3 in the nucleus, which underpins the novelty of our study. Collectively, the above data and evidence suggested that lncRNA HOXC-AS3 depletion attenuated malignant proliferation of CC cells.

Existing review has reported that lncRNAs are potent to interact with proteins so as to affect gene expression and subcellular processes (Marchese et al. 2017). Besides, several studies have demonstrated that lncRNA HOXC-AS3 promotes cell processes in cancers via binding to proteins (Zhang et al. 2018; Su et al. 2020). A case in point is that lncRNA HOXC-AS3 can decrease the ubiquitination of Y-box binding protein-1 to stabilize YBX1 by breaking the interaction between MDM2 (a E3 ubiquitin ligase) and YBX1 (Su et al. 2022). On a separate note, KDM5B has been demonstrated to be abundantly enriched in a variety of cancers (Fu et al. 2020). E3 ubiquitin ligase-mediated ubiquitination is a basic mechanism which regulates the degradation of KDM5B (Bueno and Richard 2013). Reduction in KDM5B ubiquitination mediated by PIAS4 promotes adaptation of gastric cancer cells to hypoxia (Zhou et al. 2021). In this study, KDM5B upregulation was detected in CC. Afterwards, KDM5B was proven to be a target gene of lncRNA HOXC-AS3, and lncRNA HOXC-AS3 was mainly located in the nucleus of CC cells and directly bound to KDM5B in the nucleus. Moreover, existing studies have highlighted the modulatory role of TRIM37 in the ubiquitination of both onco-proteins and tumor suppressors to affect cancer development (Chen et al. 2020; Miao et al. 2021). Subsequently, through bioinformatics analysis, we focused on E3 ubiquitin ligase TRIM37 which may promote KDM5B ubiquitination. Our experiments revealed that the TRIM37 knockdown declined KDM5B ubiquitination levels while the depletion of lncRNA HOXC-AS3 increased ubiquitination levels. Therefore, the above data indicated that lncRNA HOXC-AS3 directly bound to KDM5B and disrupted the interaction between TRIM37 and KDM5B, and therefore decreased KDM5B ubiquitination.

KDM5B functions as an oncogene to regulate multiple malignant behaviors of cancer cells. KDM5B encourages epithelial-mesenchymal transition in cancer cells and stimulates the progression of pancreatic cancer (Zhao et al. 2021b). KDM5B downregulation retards the outgrowth of gastric cancer (Li et al. 2019b). KDM5B knockdown enhances the chemosensitivity of endometrial cancer cells towards paclitaxel (Li et al. 2019a). Next, we treated CaSki cells with a combination of KDM5B upregulation and lncRNA HOXC-AS3 downregulation, after which proliferation of CC cells was strengthened. In agreement with our results, Zhou et al. (2017) has concluded that KDM5B ablation promotes cell apoptosis, hampers cell proliferation, and inactivates the Notch pathway, therefore attenuating the outgrowth of CC. Thus, these results indicated that KDM5B upregulation reversed the roles of lncRNA HOXC-AS3 knockdown in suppressing proliferation of CC cells. At last, the xenograft tumor model revealed that lncRNA HOXC-AS3 knockdown suppressed the growth of CC in vivo by limiting KDM5B expression levels.

## Conclusion

**Fig. 8:**
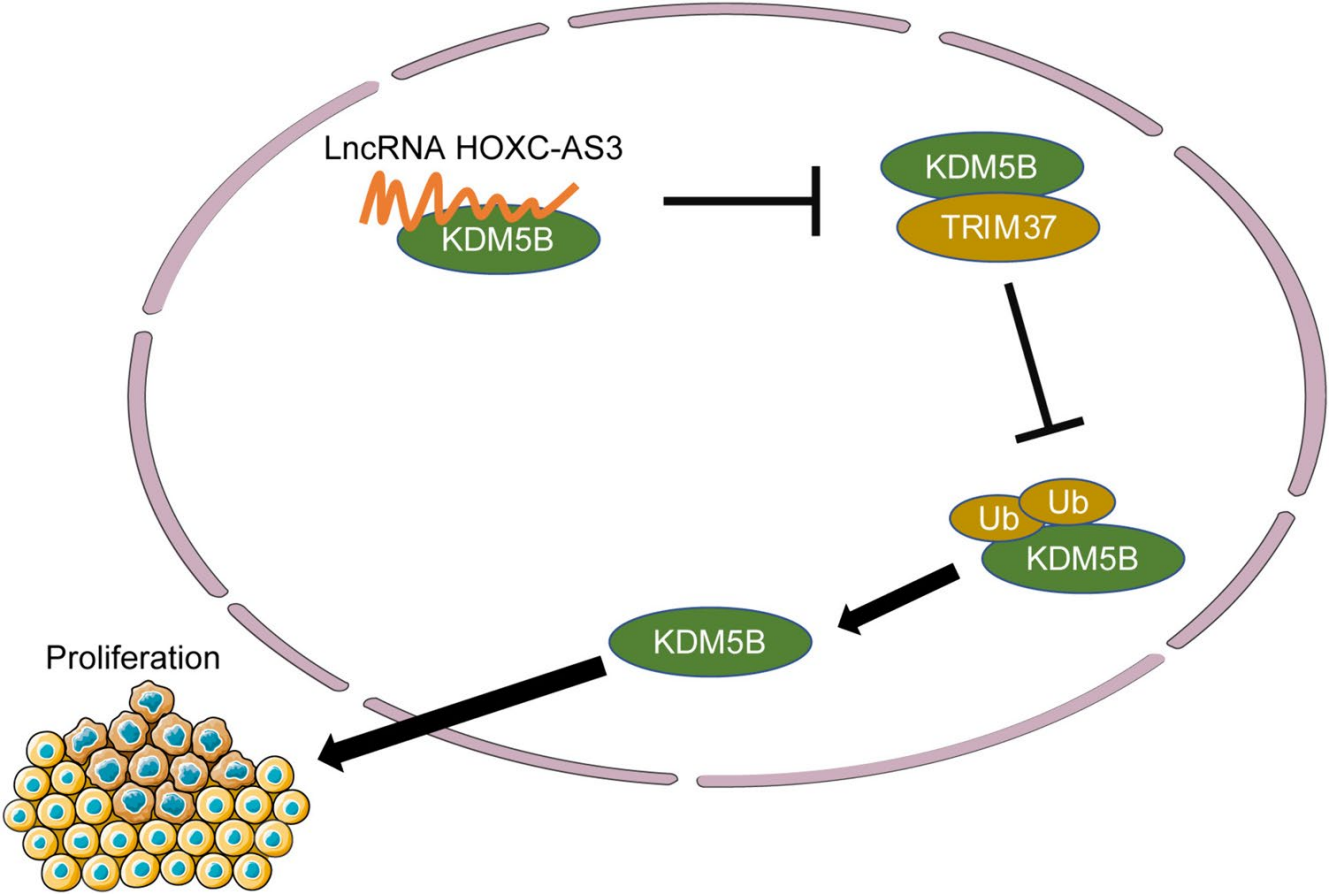
HOXC-AS3 promoted malignant proliferation of CC cells via regulating KDM5B. HOXC-AS3 directly bound to KDM5B, which inhibited TRIM37-mediated KDM5B ubiquitination and upregulated KDM5B protein levels, promoting malignant proliferation of CC cells

To sum up, the aforementioned data and results disclosed that lncRNA HOXC-AS3 binding to KDM5B repressed TRIM37-mediated KDM5B ubiquitination and upregulated KDM5B protein levels, ultimately accelerating proliferation of CC cells (Fig. 8), and this work offers an innovative insight into how lncRNA HOXC-AS3 knockdown can play a tumor-suppressive role in the progression of CC and a promising therapeutic approach against CC. However, we have yet to determine whether cytoplasm-located lncRNA HOXC-AS3 plays a role in proliferation of CC cells, seek other target proteins downstream of lncRNA HOXC-AS3, and detect the mRNA level of KDM5B in CC cells. In our next plan, we shall corroborate the role of cytoplasm-located lncRNA HOXC-AS3 in proliferation of CC cells and investigate the role of lncRNA HOXC-AS3 in other cellular functions of CC cells. Besides, it remains necessary to verify the mechanism which causes the changes in KDM5B mRNA levels and the role of this mechanism in CC, so as to expand the theoretical basis for the treatment of CC.

## Data Availability

The data used to support the findings of this study are available from the corresponding author upon reasonable request.
